# Prevalence and associated risk factors of dry eye disease in 16 northern West bank towns in Palestine: a cross-sectional study

**DOI:** 10.1186/s12886-019-1290-z

**Published:** 2020-01-13

**Authors:** Yousef Shanti, Reham Shehada, May M. Bakkar, Jamal Qaddumi

**Affiliations:** 1 Department of Ophthalmology, An-Najah National University Hospital, 44839 Nablus, Palestine; 2 Department of Medicine, College of Medicine and Health Sciences, An-Najah National University, 44839 Nablus, Palestine; 3 Department of Ophthalmology, The Islamic Hospital, Amman, Jordan; 4 Faculty of Applied Medical Sciences, Department of Allied Medical Sciences, Jordan University of Science and Technology (JUST), Irbid, 22110 Jordan; 5 Department of Nursing, College of Medicine and Health Sciences, An-Najah National University, 44839 Nablus, Palestine

**Keywords:** Dry eye disease, Prevalence, Risk factors, Palestine

## Abstract

**Background:**

Dry Eye Disease (DED) is a multifactorial disease of the interpalpebral ocular surface and tear film that leads to discomfort, fatigue and disturbance in vision. DED affects patients’ quality of life and leads eventually to decrease of productivity. Moreover, it has a considerable socioeconomic burden. It is a growing underdiagnosed health issue and the possible associated risk factors are very common and keep growing worldwide.

**Purpose:**

To assess the prevalence of DED and potential associated risk factors in the Northern West Bank of Palestine.

**Methods:**

A cross sectional study was conducted in 16 selected towns in Northern West Bank governorates during December 2016 to September 2017. An interviewer-assisted Ocular Surface Disease Index (OSDI) questionnaire was used to study DED symptoms in the study population. Further evaluation of clinical signs of DED was performed using the following objective tests: tear film break-up time (TBUT), fluorescein corneal staining (FL/S) and Schirmer test. Subjects with an OSDI score of 13 or above were considered symptomatic of DED, and DED was defined if an OSDI score ≥ 13 is accompanied by at least one of the following signs in the worse eye: TBUT ≤10 s, Schirmer score ≤ 5 mm and fluorescein corneal staining ≥ grade 1.

**Results:**

Seven hundred sixty-nine subjects were recruited from the general non-clinical population in the West Bank. The mean age of participants was 43.61 ± 18.57 years ranging from 18 to 90 years. Females constitute 52.7% of the study population. Based on the diagnostic criteria, the prevalence of DED was 64% (95% confidence interval 60.6–67.3). DED was significantly associated with female gender *p* = (0.001) and older age *p* = (0.001).

**Conclusion:**

The prevalence of DED is high in the study population. Older age and female gender were associated risk factors with the development of DED.

## Background

Dry eye disease (DED) is a common ocular surface disorder that considered a public health problem due to its impact on vision-related quality of life of the affected subjects [1, 2].According to the recent official report of the International Dry Eye Workshop (DEWS 2017), that based on summary of the findings of current research, DED was defined as “a multifactorial disease of the tears and ocular surface that is associated with hyperosmolarity of tear film which in turn leads to inflammation and damage of the ocular surface that accompanied with ocular symptoms of discomfort, fatigue and disturbance in visioin” [1].

The prevalence of DED has been reported in many countries around the world, with a range of between 9.5–90% [3–12]. This variation has been suggested to be influenced by geographical location, the variety of the population studied, variation in diagnostic criteria used with an observed lack of standard diagnostic criteria across all studied. In general, it was reported that prevalence of DED more prevalent in Asian countries compared to Western countries [13].

Epidemiologic studies showed that the disease is more prevalent among women (particularly post menopause) [14] and elderly population [15]. Additionally, a group of risk factors have been reported to be associated with DED. Those include environmental factors such as extreme temperature and reduced relative humidity [16], use of video display terminals (VDT) [17], smoking [18], refractive surgery such as LASIK [19, 20], contact lens wear [21], and uptake of certain medications such as antihistamines [22], beta-blockers [23] and oral contraceptives [6]. DED was also reported to occur with anxiety disorders, sleep disorders and depression [24, 25].

DED can be assessed based on a combination of symptoms and signs. However, several studies reported poor correlation between DED symptoms and signs [26–28]. Dryness symptoms could be assessed systematically using validated questionnaires that include questions allow for the monitoring of dryness symptoms and their frequency and or their severity over time [29]. Examples of these questionnaires include Ocular Surface Disease Index (OSDI) questionnaire, Dry Eye Questionnaire (DEQ), MacMonnies dry eye questionnaire and Standard Patient Evaluation of Eye Dryness (SPEED) questionnaire [30].

Clinical signs of DED are routinely assessed using a set of tests that include measure of tear film stability as in tear film break-up time test (TBUT), assessment of ocular surface desiccation through corneal and conjunctival staining by fluorescein or lissamine green stains, tear volume which can be estimated with Schirmer test or observation of lower tear film meniscus volume under a slit-lamp [31]. The quality of the tear film can also be assessed and monitored by measuring tear film osmolarity that based on the number of charged particles in a small tear sample measured by osmometer [31, 32].

A review of the literature showed that the prevalence of DED in the Middle East has not been frequently studied. However, according to the few studies reported in the region; the prevalence was noticeably high. For example, in the Kingdom of Saudi Arabia (KSA), DED was reported at a prevalence of 93.2% in one city on the basis of presence of one or more DED symptoms occurring often or most of the time, along with presence of one or more of the dryness signs revealed by clinical tests [33]. In another recent study in KSA but in a different region (Al-Ahsa) the prevalence of dry eye disease was reported at 32% based on presence of one or more of DED symptoms in a frequency of often or constantly [34]. In a cross sectional study that conducted in Jordan and based on OSDI questionnaire, it was reported that 59% of the study population reported an OSDI score equal or greater than 20 and accordingly considered DED symptomatic [3]. In Iran, a study was conducted among 40–64 years old population in one city reported a prevalence of DED of 8.7% based on an OSDI score of 23 or higher and presence of one positive sign of DED [4].

The prevalence of DED in Palestine has not been studied before. Therefore, this study aims to study the prevalence of DED and to investigate possible risk factors of the disease in a general non-clinical population in Palestine.

## Methods

### Study population

Multistage sampling method -based on the Palestinian central bureau of statistics sampling frame- was used to identify the Palestinian towns participating in the study. Sixteen towns in Northern six West Bank governorates were randomly selected to enroll in the study. Participants were randomly selected and invited from phone directory of 16 towns in Northern six West Bank governorates to attend a free medical event held in municipal councils of their towns. The maximum number of participants who fit the study inclusion criteria was 900. However, the final number of respondents who were able to attend the free medical day was 769 from 16 towns in Northern West Bank governorates.

The needed number of participants depended on the percentage of Palestinians in each city. Given that the number of Palestinians in the selected cities in the west bank is about 391.821 people, a minimum complete data from 384 participants would be needed. Using http://www.raosoft.com/samplesize.html, this would allow the percentage of correct answers to be estimated with a 95% confidence interval and margin-of-error of at most ±5%. So, 769 participants thought to be adequate to enrol in the study.

The cross-sectional study was conducted between September 2016 and January 2017.Only subjects aged 18 and above were interviewed for the study.

### Exclusion criteria

Exclusion criteria included patients who are contact lens wearers, those who underwent refractive surgery procedures and those with an active ocular surface disease and any other conditions that may interfere with development of DED. Subjects with previous diagnosis of DED were included in the study.

### The questionnaire

Participants were interviewed in using the Arabic version of the OSDI questionnaire by trained surveyors. A pilot study revealed that the Arabic questionnaire was easily administered, socio-culturally acceptable and understandable [3].

The participants asked to rate their own ocular symptoms over the preceding week. Each time a participant indicated the existing of dry eye symptom, he/she was further asked to grade its frequency on a scale of 0 to 4, where 0 indicates none of the time; 1, some of the time; 2, half of the time; 3, most of the time; and 4, all of the time. To calculate the OSDI score, total points of patient’s responses were multiplied by 25 and then divided by the total number of responses [35].

Additionally, other demographic data (age, gender), medical and ocular history were acquired from each participant. The risk factors evaluated were age, gender, VDT use and smoking.

### Examinations

A sequence of ocular examinations was performed for each participant. Those include best corrected visual acuity which was assessed using Snellen visual acuity chart, manifest refraction using retinoscopy. Slit-lamp biomicroscopy was used for assessment of adnexa and ocular surface health, corneal staining and dry eye testing.

Dry eye objective tests were performed in the following order: measurement of fluorescein-assisted tear film break-up time TBUT, meibomian glands evaluation and Schirmer test. TBUT was performed by fluorescein strip that was slightly wetted with non-preserved saline and administered to the lower conjunctiva. TBUT was then determined by counting the time in seconds between the last blink and the appearance of the first dry spot in the pre-corneal tear film observed by slit-lamp with blue light filter. This process was repeated three times for each eye and an average of the three values was recorded for each eye. Level of staining on the cornea was graded using Efron scale [31].

Schirmer test was performed without anesthesia using a sterile Schirmer strip that was placed at the junction of the lateral one third to medial two thirds of the lower lid and left for 5 min while the patient blink normally. The length of wetted Schirmer strip was recorded in millimeters.

For all given dry eye objective tests used in this study, both eyes were examined and data was recorded for both eyes. In cases of variation between the two eyes, data from the worse eye was used for statistical analysis.

### Diagnostic criteria

Presence of DED was investigated through evaluation for DED symptoms and clinical signs. DED was defined using diagnostic criteria recommended by DEWS 2017 report [1, 36]. DED diagnosis was confirmed by the presence of subjective DED symptoms revealed with an OSDI score of ≥13 which is accompanied by at least one of the following objective DED signs in the worse eye: TBUT ≤10 s, Schirmer score ≤ 5 mm and fluorescein corneal staining ≥ grade 1 [4, 37, 38].

### Statistical analysis

Data were analyzed using the statistical Package for Social Sciences (SPSS) software version 21.0 (SPSSJ: Inc., Chicago, IL, USA). Chi-square test was applied on the categorical variables. Prevalence of DED in the studied population and confidence intervals (CIs) were calculated for risk factors. Odds ratio and associated risk factors with the DED were evaluated using bivariate and multivariate logistic regression analysis at 95% CI. A *p*-value of less than 0.05 was considered statistically significant.

## Results

### Participant characteristics

The study was conducted during December 2016 to September 2017. A total of 769 participants were recruited from six governorates in Northern West Bank. The mean age of all participants was 43.61 ± 18.57 years with a range of 18 to 90 years. Of all study population, 405 (52.7%) subjects were females and 364 (47.3%) were males. Of the study population, 131 (17%) reported having diabetes milletus (DM), 161 (20.9%) reported having hypertension (HTN).

The demographic characteristics of the study population are shown in Table 1**.**

**Table 1:** participants’ characteristics (*n* = 769)

Variable	No. (%) of respondents
Gender	
Male	364 (47.3)
Female	405 (52.7)
Age group(yrs)	
18–25	179 (23.3)
26–45	225 (29.3)
> 45	365 (47.5)
Smoking	
Non-Smokers	542 (70.5)
Smokers	227 (29.5)
Education level	
Illiterate	67 (8.7)
High school	481 (62.5)
Higher education	221(28.8)
Residence	
Jenin	325 (42.26)
Tulkarem	158 (20.5)
Nablus	150 (19.5)
Tubas	56 (7.3)
Qalqilia	42 (5.5)
Salfit	38 (4.9)

### Prevalence of dry eye disease

Based on the DED diagnostic criteria clarified in the methodology section; of an OSDI score ≥ 13 and presence of one or more clinical signs of DED, the overall prevalence of DED was 64%(95% confidence interval 60.6–67.3) in the study population. The average age of patients with DED was 45.24 ± 18.8 years. Table 2 shows the distribution of DED by age groups and gender.

**Table 2:** distribution of definite dry eye disease (DED) by age group and gender

	DED					
Age groups (years)	Yes			No		
	Male (n)	Female (n)	Total (n)	Male (n)	Female (n)	Total (n)
18–25	45	62	107	41	31	72
26–45	57	70	127	53	45	98
>45	105	153	258	63	44	107
Total (n)	207	285	492	157	120	277

DED was significantly more prevalent in older age group > 45 years (*p* = 0.002) and in females than males in all age groups (*p* = 0.00).

The prevalence of DED (an OSDI ≥13 and presence of at least 1 clinical sign) and its signs are summarized in Table 3. From the overall study population of 769 subjects, the proportion of subjects with an OSDI score equal or greater than 13 (the cut off value) was 71%.The most prevalent DED sign is abnormal TBUT (≤ 10 s) as it is revealed among males and females within all age groups. Whereas, Schirmer test score of ≤5 mm was the least prevalent sign among all the study population.

**Table 3:** The prevalence (%) of DED and its signs and symptoms by age and gender. Data are presented for the worse eye only

	N	OSDI score ≥ 13	TBUT ≤ 10 s	Schirmer test ≤ 5 mm	Corneal fluorescein staining ≥ 1	DED
		N (%) (95% CI)	N (%) (95% CI)	N (%) (95% CI)	N (%) (95% CI)	N (%) (95% CI)
Age groups						
18–25	179	107 (60%)	123 (68.8%)	10(5.6%)	90 (50.4%)	107 (59.8%)
		(52.5–66)	(64–73.3)	(2.2–9.2)	(43.4–56.5)	(52.5–66.7)
26–45	225	126 (56.2%)	170 (75.6%)	22 (9.8%)	132(59.1%)	127 (56.4%)
		(50–63.6)	(73–77.9)	(6.1–14.8)	(52.9–64.1)	(50.2–63.6)
>45	365	256 (70.8%)	270(74.2%)	39(0.7%)	273 (75.1%)	258 (70.7%)
		(65.7–74.7)	(71.5–76.4)	(7.7–14)	(72.9–77.2)	(66–75.3)
Male	364	205 (56.5%)	185 (51%)	19 (5.2%)	232 (64.1%)	207 (57%)
		(51.8–60.9)	(48.2–54)	(2.8–7.4)	(60.2–67.6)	(52–61.5)
Female	405	286 (70.8%)	223 (55.3%)	52 (12.8%)	264 (65.2%)	285 (70.4%)
		(66.4–75.3)	(53.3–57.3)	(9.9–16.3)	(62–68.9)	(66.4–75.1)

### OSDI score results

The calculated mean OSDI score for all participants was 27.7 ± 21.4.The mean OSDI score in the definite DED group was 37 ± 19.For the non-dry eye group, the average OSDI score was 11 ± 14.

From the overall study population of 769 subjects, the proportion of subjects with an OSDI score equal or greater than 13 (the cut off value) was 71%. The frequency of different levels of dryness symptoms severity based on OSDI score is shown in Table 4**.**

**Table 4:** Severity of DED symptoms based on OSDI score

Level of OSDI score	N (%)
Normal (0–12)	224 (29.1)
Mild (13–22)	150 (19.5)
Moderate (23–32)	122 (15.9)
Severe (33–100)	273 (35.5)

### DED objective signs

In definite DED group the mean TBUT was 5 ± 2.4 s, the mean Schirmer test value was 17.4 ± 8.7 mm.

### Risk factors of dry eye disease

The risk factors associated with the definite DED were evaluated using a bivariate and a multivariate logistic regression analysis. Significant odds of having DED were associated with older age (older than 45) and female gender **(**Table 5**).**Whereas, definite DED was not significantly associated with smoking and presence of systemic disease.

**Table 5:** logistic regression analysis of risk factors associated with definite DED

Risk factors	Definite DED (n)	*P* value	Adjusted OR (95% CI)
Age (years)		0.001*	1.018 (1.008–1.029)
18–25	107		
26–45	127		
> 45	258		
Gender		0.001*	0.524 (0.364–0.755)
Male	207		
Female	285		
VDT use (yes vs. No)	208	0.231	1.040 (0.976–1.108)
Smoking	132	0.854	0.964 (0.652–1.426)
Systemic disease			
DM	88	0.841	1.047 (0.669–1.637)
Hypertension	108	0.433	1.190 (0.771–1.836)

*OR* Odds ration, *DED* Dry eye disease, *CI* Confidence interval, *VDT* Video display terminal, *DM* Diabetets milletus

* represent a *p* value of less than 0.05 which is considered statistically significant

The average daily usage of VDT in the DED and non-DED groups was 1.9 and 1.6 h, respectively. However, VDT usage was not significantly correlated with the development of DED.

## Discussion

Few studies have investigated the prevalence of DED in the Middle East. This is the first population-based study regarding prevalence and risk factors of DED in Palestine. The study combined both subjective and objective clinical tests to confirm the diagnosis of DED based on the diagnostic criteria recommended by latest DEWS guidelines published in 2017 [1]. Associated potential risk factors with the development of DED were also evaluated.

In the study, the OSDI questionnaire was used to assess the presence of symptomatic DED. Patients also underwent an assessment of the DED clinical signs those include corneal staining, Schirmer test and FTBUT. DED was confirmed by presence of dryness symptoms and at least one clinical sign.

The data reveals that DED is a prevalent disease in west Palestine; where 69% of the study population reported symptoms of the disease and manifested DED clinical signs. This prevalence is higher than other reports in the region with similar arid climate [3, 34]. The high prevalence might be attributed to several factors mainly related to the arid climate with temperature ranging from 30to 40 °C during summer seasons.

Furthermore, the psychological aftermaths of the Palestinian-Israeli Conflict which was suggested as affecting the Palestinians in a wider range could have increased the levels of stress and anxiety among this population [39]. The psychological aftermaths of the conflict and violence including posttraumatic stress disorders could also have strong public health impact [39], and according to the ophthalmic literature, individuals with anxiety and stress, with or without psychiatric disease, are more expected to experience DED [24, 25, 40].

The prevalence of DED varies widely between different population-based studies, which make it difficult to compare findings from these studies. The variability in prevalence of DED may contribute to the type of targeted population (clinical or non-clinical), studied population’s age group and differences in DED diagnostic criteria that may rely on subjective reported symptoms detected by questionnaire or usage of objective clinical tests. Table 6 summarizes results from different studies around the world on the prevalence of DED.

**Table 6:** prevalence of DED from different studies around the world

Country/area	Sample number	Prevalence	Diagnostic criteria	Age group (yrs)	Authors (year)
This study/ Palestine	769	69%	One or more symptoms often or all most thetime, accompanied by at least one sign (TBUT, Schirmer test and FL/S of the cornea)	18–90	
Jordan	1039	59%	Symptoms only using OSDI questionnaire, a score of 20 or above considered symptomatic DED.	≥18	Bakkar et al. (2016) [3].
Saudi Arabia (Jeddah)	251	93.2%	One or more symptom often or all most time, accompanied by at least one signs (TBUT, Schirmer test and FL/S of the cornea	7–78	Bukhari et al. (2009) [33].
Saudi Arabia (Al-Ahsa)	1858	32.1%	6-item questionnaire. DED is determined by presence of one or more of the six DES symptoms often or constantly.	16–78	Alshamrani et al. (2017) [34].
Iran, Shahroud	1008	8.7%	Symptoms using OSDI questionnaire and presence of at least one of objective signs (Schirmer test, TBUT, fluorescein and rose Bengal staining).	40–64	Hashemi et al. (2014) [4].
United States/female population only	39,876	7.8%	in the presence of either a prior clinical diagnosis of DES or intense symptoms (both irritation and dryness whether many times or all the times)	45–84	Schaumberg et al.(2003) [41].
United States/ Hispanic population	463	43.6%	One simple question about symptomatic dryness was asked. “How often do you have dryness?” The answers were forced choice: never, seldom, sometimes, frequently, or always.	4–85	Hom et al. (2005) [42].
USA, Wisconsin	3722	14.4%	Self-recorded history of DED through the previous 3 months	48–91	Moss et al. (2000) [6].
USA, Maryland	2420	14.6%	One or more dry eye symptoms often or all the time (six items) Meibomian glands assessment, rose Bengal, Schirmer’s test.	65–84	Schein et al. (1997) [43].
Indonesia/Sumatra	1058	27.5%	One or more symptoms many times or all the time using a six item validated questionnaire.	≥21	Lee et al. (2002) [5].
Japan/Tokyo	598	33%	Self-administered Questionnaire	20–49	Shimmura et al. (1999) [44].
Japan	113 (Left eye only)	73.5%	Japanese diagnostic criteria of dry eye (Schirmer test, TBUT, FL/S of the cornea)	≥ 60	Uchino et al. (2006) [45].
Australia	1174	57.5%	Dry eye questionnaire	≥50	Chia et al. (2003) [46].
Australia	1584	10.8%	McMonnies dry eye questionnaire, TBUT and Rose Bengal ocular surface staining	3–96	Albietz et al. (2000) [47].
Australia/Melbourne	926	16.3% by (Schirmer’s) 8.6% by (TBUT) 1.5% by (FL/S) 10.8% by (rose Bengal) 5.5% with any sever symptom	Objective assessment Schirmers< 8, TBUT <8, FL/S, rose bengal> 3 and intense symptoms (3 on a scale of 0 to 3)	40–97	McCarty et al. (1998) [48]
Taiwan, Taipei	1361	33.7%	Reporting one or more dry eye symptoms often or all of the time.	≥ 65	Lin et al. (2003) [49].
Thailand, Bangkok	550	34%	One symptom or more many times or most of the time, TBUT, Schirmer’s test, FL/S, assessment of Meibomian gland	40–78	Lekhanont et al. (2006) [50].

In this study, definite DED diagnosis was more prevalent in subjects older than 45 years. This in agreement with many reports that found age related to DED development [3, 4, 33, 34]. The results also showed an association between DED diagnosis and female gender as females have 1.5 times higher risk of developing DED compared to males. This finding could be explained by use of hormones for contraception or infertility in the younger women age group and the impact of these hormones on the female’s lacrimal gland, goblet cell function, MG and ocular surface sensitivity that may contribute to dry eye symptoms [11]. In women within the older age group, lower levels of estrogens and androgen may lead to inadequate lacrimal gland secretion that associate with aqueous deficient DED [51].The impact of gender on the development of DED varies across studies. Consistent with the current study, most studies reported that DED occurs more likely among females [3, 4, 6, 34, 46, 48, 49].

The results of this study showed no association between DED and smoking habit. This finding is in agreement with other reports where smoking was not associated with the development of DED [3]. However, few studies reported an association of smoking with DED [6, 34].

There was also no association between DED and VDT usage.This findingcontradicts with many published reports where VDT plays a role with development of DED [10, 52]. In the current study, this may explained by that 47.5% of the study population are within the older age group (> 45 years), where VDT usage is more common in younger population.

The current study showed that a high number of subjects had tear-film instability that characterized by abnormal TBUT of less than 10 s and corneal staining of a grade 1 or higher which was observed in 93%, 84%of subjects, receptively. Whereas, the least reported sign was Schirmer score of less than 5 mm. This may suggest that DED in the studied population might affect the tear film quality or instability rather than the tear film quantity. However, Schirmer’s score might be underestimated sign due to irritation caused by the Schirmer’s strip that may induce reflex tearing which could mask the sign of reduced tear quantity on the ocular surface. It was also suggested in the literature that Schirmer scores have poor correlation with dry eye symptoms [53].

From the overall study population, 71% had an OSDI score of 13 or greater; a proportion higher than the prevalence of definite DED in this study. Depending only on the OSDI scores or presence of dryness symptoms to diagnose DED may result in an overestimation of the DED in the studied population. This emphasizes the need to combine both clinical signs and symptoms to define DED [54].

The study may have some limitations. The exclusion of contact lens wearers, post refractive surgery patients and patients with severe conjunctival and corneal disease might underestimate the actual prevalence of DED in the studied population. Additionally, meibomian gland dysfunction and tear film osmolarity tests were not assessed in the study population, while these tests play an important role in the accurate diagnosis of DED.

Finally, FTBUT was used in the current study. However, non-invasive BUT was recommended by DEWS II to use as it is longer than FBUT in general. However, in the same DEWS II 2017 report it was stated that “measurement of the tear breakup time with a noninvasive technique (NIBUT) is considered preferable to the FBUT and the two techniques are well correlated” [55].

## Conclusion

In conclusion, this is the first study of the prevalence of DED in a previously unstudied population in Palestine. It was found that the prevalence of DED is relatively high in this study. An attempt should be performed to increase the awareness of the society with DED, so modifiable risk factors can be reduced. However, further research is required to better understand other potential risk factors associated with DED including; impact of arid environment, drug use, systemic diseases and anxiety.

## Data Availability

All data supporting the study is presented in the manuscript or available upon request from the corresponding author of this manuscript.

## Abbreviations

CI: Confidence intervals
DED: Dry Eye Disease
DEQ: Dry Eye Questionnaire
DEWS 2017: International Dry Eye Workshop
FL/S: Fluorescein corneal staining
IRB: Institutional Review Board
KSA: Kingdom of Saudi Arabia
OSDI: Ocular Surface Disease Index
SPEED: Standard Patient Evaluation of Eye Dryness
SPSS: The statistical Package for Social Sciences
TBUT: Tear film break-up time
VDT: Video display terminals
